# Development of Educational Print Materials for Physical Activity in Cancer: Evaluation of Readability and Suitability

**DOI:** 10.1007/s13187-021-02076-1

**Published:** 2021-09-15

**Authors:** Alice Avancini, Giulia Benato, Daniela Tregnago, Ilaria Trestini, Michele Milella, Massimo Lanza, Sara Pilotto

**Affiliations:** 1 Medical Oncology, University of Verona, Verona, Italy; 2 Department of Neurosciences, Biomedicine and Movement Sciences, University of Verona, Verona, Italy

**Keywords:** Patient educational booklet, Suitability assessment, Physical activity promotion, Exercise, Cancer

## Abstract

Educational health materials may be important tools to increase physical activity in cancer patients. Nevertheless, most of the available resources regarding physical activity for cancer patients were found not suitable, had a low grade of readability, and thus, represent a significant barrier to behavior change. To date, little data about development criteria and evaluation of physical activity resources for cancer before their spread exist. The purposes of this study were (i) to describe the development of a physical activity guidebook designed for cancer patients and (ii) to test its readability and suitability. The guidebook was developed through multi-step passages, including group discussions, a literature review, identification of a motivational theory, and using previous research on exercise preferences, barriers, and facilitators to target the information. Two validated formulae were used to assess the readability, whereas thirty-four judges completed the Suitability of Assessment Materials questionnaire to evaluate the suitability of the guidebook. The guidebook was found readable for patients having at least a primary education, and the judges scored it as “superior” material. Our guidebook, following a rigorous method in the development phase, was considered to be suitable and readable. Further evaluations through clinical trials could investigate its effectiveness for behavior change and its impact on cancer patients.

## Introduction

Nowadays, oncological diseases represent an important burden on societies worldwide. In Italy, it is estimated that about 1030 new cancer cases per day were diagnosed in 2020 [1]. Increasing knowledge of molecular and tumor biology has changed cancer treatment paradigms notably over the past two decades, resulting in an increased number of survivors [2]. On the other hand, antineoplastic therapies may cause various side effects, such as fatigue, pain, and cardiovascular disorders that could negatively affect patients’ quality of life. In this light, supportive care like physical activity (PA) may improve treatment response and, at the same time, reduce the toxicity burden [3]. From this point of view, a growing amount of literature reports that PA is effective in cancer prevention, controlling disease progression, and potentially improving anticancer treatment response [2]. Nevertheless, and unfortunately, a large number of cancer patients still remain insufficiently active [4].

In this light, developing and validating new methods promoting PA in the cancer population represent an urgent and unmet clinical need. Written PA materials, guidebooks, pamphlets, and booklets may be an optimal strategy to encourage people to increase PA, as well as informing patients about the associated risks and benefits of this behavior, at a relatively modest cost [5–7]. Moreover, they may have a large number of advantages such as the consistency of message, reusability, portability, and usability to supplement or reinforce verbally acquired information, maximizing patients’ knowledge and adherence to treatment by refreshing their memory [8]. In the oncological population, written health materials showed effectiveness in inducing PA behavior change and consequent benefits from this change. For instance, a recent systematic review evaluating PA intervention among colorectal cancer survivors found that written materials delivered by e-mail were able to improve PA quality of life and reduce the recurrence risk [9]. In a randomized controlled trial, Vallance and colleagues investigated the effects of print materials and pedometers on 377 breast cancer survivors. Compared to standard PA recommendations, cancer-specific PA print materials combined with step pedometers significantly increased total PA, improved patients’ quality of life, and reduced fatigue [10].

However, design and written health information materials require several aspects to be considered, to develop a usable resource and maximize the effectiveness of the message [8]. To support a lifestyle change, the researchers advocate that written resources should be theoretically based so as to trigger a patient’s motivation in order to implement the behavior. In this sense, a variety of constructs have been investigated in the context of “exercise oncology” and show that the inclusion of motivational theories into planning a PA intervention may enhance the beginning, the adherence, and the maintenance over time of an active lifestyle [11]. Although written materials have demonstrated to be effective, almost one in two citizens has limited health literacy, i.e., the capacity to obtain, process, and understand health information and services to make sound health decisions, which translates into a scarce ability to understand information and use it to make appropriate decisions regarding their health [12]. In this context, educational print materials in order to be effective should conform to readability standards, i.e., the reading difficulty of a resource, and suitability, i.e., how well such material is considered appropriate [8, 11]. Unfortunately, few materials meet these standards. For example, previous data reported that written health information is often unsuitable and has insufficient readability, thereby limiting its efficacy [13, 14]. For these reasons, written educational materials should be developed according to these factors and tested for suitability and readability before their release to optimize the possibility of modifying behavior that involves the principal stakeholders of the resource [15].

As regards PA, to our knowledge, only two studies reported the development stage, assessed suitability, readability, and appropriateness of written health information in cancer patients [16, 17], and none was in the Italian cancer population. The purpose of this study was to develop and evaluate the suitability and readability of “*Informa*” (“inform” in English), a PA guidebook specifically designed for cancer patients.

## Materials and Methods

This study utilized a cross-sectional design. Data were collected at the University of Verona, Italy, between April 2020 and December 2020. The project was reviewed and approved by the Ethics Committee for Clinical Trials (Prot. N. 67,002), University of Verona. All study procedures were conducted following the latest revision of the declaration of Helsinki, as well as the declaration of Oviedo. The study protocol was designed to adhere to Good Clinical Practice principles and procedures and complied with Italian legislation. All the included participants signed the written informed consent.

### Development of the Informa Guidebook

**Fig. 1:**
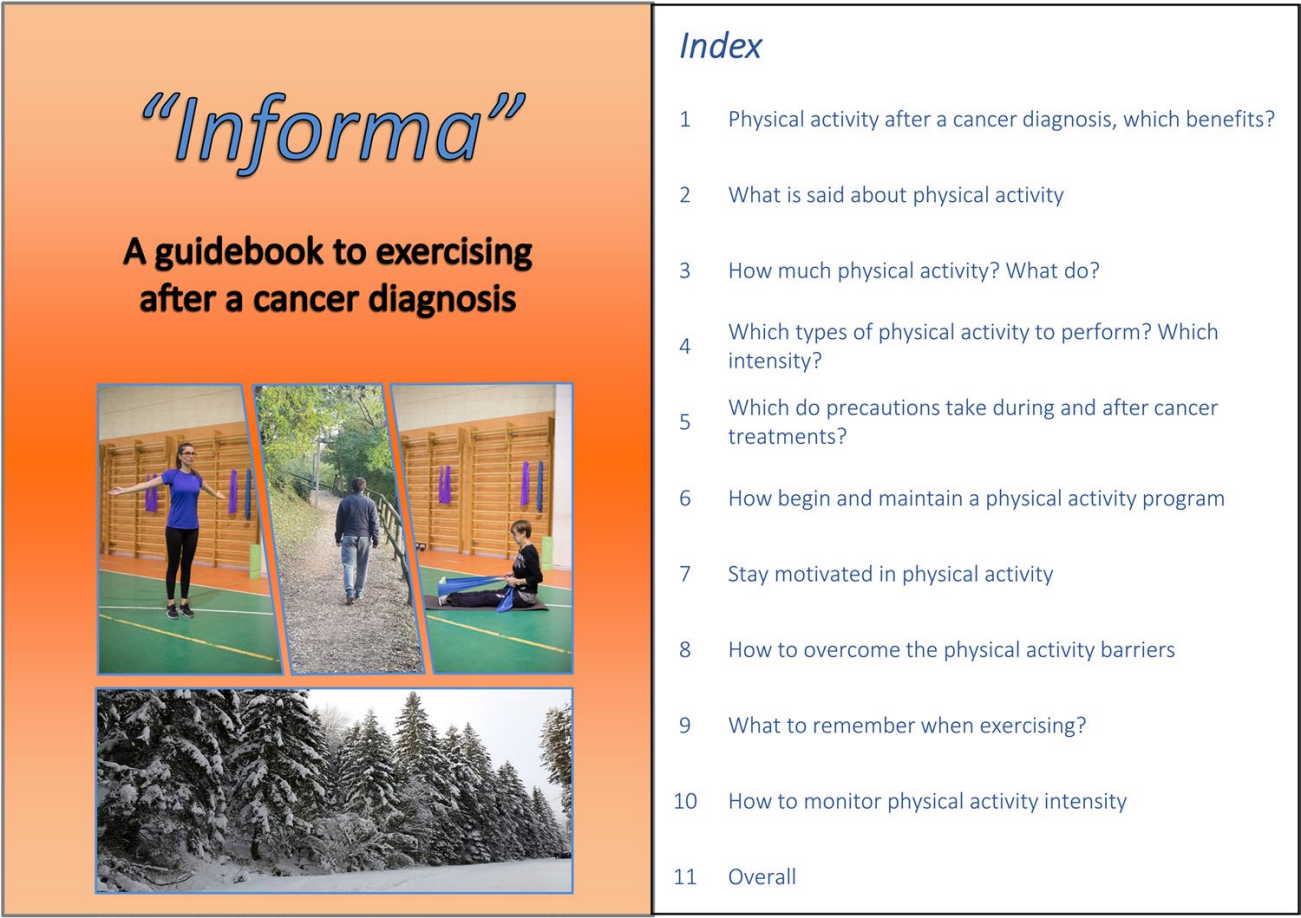
*Informa* guidebook cover image and index

The *Informa* guidebook is written in Italian, on A5 paper size (14.8 × 21 cm), and consists of 11 chapters (Fig. 1), with the aim of improving PA promotion in oncological settings. Its development is the result of several steps, as outlined in Arora [12] and Hoffmann [15]. Firstly, a group discussion between the members of the research team was carried out to clarify the main points about how to create the guidebook, who the target audience was, as well as to discuss general recommendations for designing effective health education materials. Afterward, team members, AA and GB, performed an in-depth literature review to detect the key findings in exercise-oncology, propose an evidence-based guidebook, and the information about how to write a useful written health material. Specifically, the benefits of PA with cancer, the current exercise guidelines, the risks, and specific precautions to adopt during exercising all oriented the research.

Moreover, to target the included information better, the *Informa* guidebook was also based on our previous studies, examining cancer patients’ preferences about exercise and the features that potentially hindered or triggered patients to increase PA [4, 17]. As suggested by the literature, the third step consisted of identifying an appropriate theory to orient the overall development and drafting of the *Informa* guidebook to stimulate patient motivation efficaciously. The Theory of Planned Behavior was effective and widely applied to enhance PA in the cancer context [11]. This theory postulates that behavior is predicted by intention. In turn, intentions are directly predicted by attitude, i.e., the perception of behavior as positive or negative, by subjective norms, i.e., the perceived normative belief of others in the behavior, and by perceived control, i.e., the belief that a behavior can be performed with easiness or difficulty [11]. After these multi-steps, another group discussion among the research team members was performed to solve doubts and concerns and reach a consensus about the points to guide the guidebook’s development. AA wrote the first draft of the *Informa* guidebook, following the recommendations for suitability and readability proposed by Hoffmann and Worral [15]. After a revision by the research team and successive adaptations according to the team’s comments, the last draft was approved (Fig. 1), and 50 copies of the 62-page guidebook were initially printed. The next step was to test the guidebook’s readability and suitability.

### Participants and Procedures

Key stakeholders, including exercise specialists, cancer patients, and oncology healthcare providers, were included in the study to assess the readability and suitability of the *Informa* guidebook. Whereas cancer patients were chosen for their familiarity with the covered population to address the guidebook, the other evaluators were selected as experts in the field of their professional experience and skills regarding the discussed topic. All the potential participants were first contacted by e-mail to explore their interest in participating in the study. If interested, printed copies of the *Informa* guidebook and the questionnaires, with brief instructions, were delivered to the participants.

### Measures

General characteristics of the participants were obtained using a questionnaire that included age, sex, and cancer-related information (for patients only), i.e., cancer site, stage of the disease, time from diagnosis, cancer treatments, and work information (for experts), i.e., type of work, years in practice, and area of interest.

The suitability of the *Informa* guidebook was evaluated using the Suitability Assessment of Materials (SAM). The SAM is a validated tool created by Doak et al. [18] and is designed to assess printed health-educational materials. The application of SAM can pinpoint specific deficiencies in suitability, and if the material is still in the development stage, the deficiencies can be corrected. The SAM considers 22 characteristics representing six factors: content (purpose, content topics, scope), literacy demand (reading level, writing style, sentence construction, vocabulary), graphics (front page graphics, type of graphics and illustrations, relevance of illustrations, captions used for graphics), layout and type (subheadings, typography, layout), learning stimulation and motivation (interaction used, desired behavior patterns modeled), and cultural appropriateness (logic, language, experience, cultural image and examples). Each item is rated as 2- “superior”, 1- “adequate”, or 0- “not suitable”, according to the relative difficulty of decoding the words and the relative difficulty of understanding the meaning. The sum of the obtained ratings is divided by the total possible score and transformed into a percentage. Three levels are used to rate the percentage score: 70–100% “superior”, 40–69% “adequate”, and 0–39% “not suitable” [18]. Unfortunately, an Italian version of the SAM questionnaire is not available. Thus, SAM was translated in Italian by a native translator, who was familiar with the specific terminology (forward translation). Thereafter, a second native translator, without knowledge of the questionnaire, translated the document back to English (backward translation). Discrepancies between forward and backward translations were discussed by the two translators, and doubts were resolved with the study team members (AA, ML, SP, DT). Additionally, after the conclusion of the SAM questionnaire, an optional space to give written qualitative feedback was provided.

To measure readability, two different indexes, specific for the Italian language, were proposed. The Flesh-Vacca Index, i.e., the adaptation of the Flesh-Kinkaid for English, evaluates readability based on the number of syllables in the first 100 words and the average of words per sentence, through the following formula: readability = 206 — (0.65 × number of syllables in the first 100 words) — number of words in each sentence/total number of sentences [19]. The Flesh-Vacca Index grades the readability ease on a scale from 0 to 100, where a lower score corresponds to a higher difficulty (0–50 requires a “secondary education”, 50–80 requires a “primary education”, whereas “no education” is requested for a score above 80) [19].

The GulpEase index was also used to assess readability. This index is designed and built around the Italian language and has the advantage of avoiding the syllable count because it is considered unsuitable for the Italian language. Indeed, GulpEase is based on the average number of characters per word and words per sentence, according to the formula: readability = 89 — ((number of characters × 100/total number of words)/10) + 3 × ((number of sentence × 100/total number of words)) [20]. The GulpEase score ranges from 0 to 100, where a lower score corresponds to a higher difficulty of readability: 0–59 requires a “secondary education”, 60–79 requires a “primary education”, and above 80 requires “no education” [20].

### Analyses

A descriptive statistic was applied. General characteristics of the participants were analyzed using mean and standard deviation, for continuous variables, and absolute frequencies, for categorical variables. Results of overall and for each domain of suitability are presented as average. If the readability resulted ≤ 50 for Flesh-Vacca score or < 60 for GulpEase and the obtained suitability was not “superior”, i.e., ≥ 70, a revision and adjustment of the guidebook and consequently a new evaluation of these measures was provided. Data analysis was performed with STATA statistical package, version 14 (Stata Corp, Texas, TX, USA).

## Results

Participant characteristics are listed in Table 1. Thirty-four experts were enrolled in the validation of the study: (a) cancer patients (*n* = 10), (b) oncologists (*n* = 13), (c) a biologist (*n* = 1), (d) a nurse (*n* = 1), (e) researchers in physical activity (*n* = 7), and (f) kinesiologists (*n* = 2).

**Table 1:** General characteristics of the study participants

Variable	Number (percentage)
Sex	
Male	8 (24%)
Female	26 (76%)
Education	
Secondary (up to 14 years)	6 (18%)
Secondary (up to 18–19 years)	4 (12%)
College/university	16 (47%)
Postgraduate	8 (24%)
Work^a^	
Medical oncologist	13 (54%)
Researchers in physical activity	7 (29%)
Kinesiologist	2 (8%)
Biologist	1 (4%)
Nurse	1 (4%)
Years in practice^a^	
0–5	10 (42%)
5–10	4 (17%)
10–15	6 (25%)
> 15	4 (17%)
Tumor site^b^	
Breast	4 (40%)
Upper gastrointestinal tract	3 (30%)
Colorectal	1 (10%)
Lung	1 (10%)
Genitourinary tract	1 (10%)
Disease status^b^	
In remission/cured	2 (20%)
Early	4 (40%)
Advanced	1 (10%)
Metastatic	3 (30%)
Treatments^b^	
Surgery	5 (50%)
Chemotherapy	10 (100%)
Radiation therapy	3 (30%)
Hormone therapy	2 (20%)
Other	2 (20%)
Treatment status^b^	
Ongoing	10 (100%)

^a^Questions not addressed to cancer patients

^b^Question addressed only to cancer patients

With regards to readability, the GulpEase score was 61, whereas the obtained Flesch-Vacca index was 66. According to these points, the *Informa* guidebook was readable by persons who completed primary education.

The *Informa* guidebook reached an overall 94% score in the SAM questionnaire (Table 2) and was classified as a “superior” material, according to the abovementioned criteria (Table 2). In an analysis of the six subcategories (Table 2), they all had a score of ≥ 70. Specifically, the contents of the materials were rated suitable at 94% for experts and at 100% for patients, whereas the literary demand, the assessment of the reading level, writing style, sentence construction, vocabulary, and organizers, was scored suitable by both the experts (94%) and the patients (99%). The graphic category was considered suitable overall (88%), with the single item of the cover graphic scored as “adequate”. The category of layout and typography, learning stimulation and motivation, as well as cultural appropriateness, showed similar trends for experts and patients. No written feedback about potential modification in the guidebook was provided.

**Table 2:** Percentage of SAM scores according to the six covered area

Criteria	All (*n* = 34)	Experts (*n* = 24)	Patients (*n* = 10)
Content	96	94	100
Purpose is evident	99	98	100
Content about behaviors	97	96	100
Scope is limited	97	96	99
Summary or review included	93	90	100
Literacy demand	95	94	99
Reading grade level	96	94	100
Writing style, active voice	97	98	95
Sentence construction	91	88	100
Vocabulary	99	98	100
Advance organizers	94	92	100
Graphics	88	85	96
Cover graphic shows purpose	62	54	80
Type of illustrations	99	98	100
Relevance of illustrations	93	90	100
Lists and tables explained	97	96	100
Captions used for graphics	90	85	100
Layout and typography	97	96	100
Typography	96	94	100
Layout	100	100	100
Subheadings and “chunking”	96	94	100
Learning stimulation and motivation	94	92	100
Interaction included in text/or graphics	93	90	100
Behaviors are modeled	94	92	100
Motivation	96	94	100
Cultural appropriateness	95	93	98
Cultural match-logic language, experience	90	85	100
Cultural image and example	91	90	95
Overall	94	92	99

## Discussion

The purpose of this study was to describe the development and to test the readability and suitability of *Informa*, a guidebook for physical activity in cancer. *Informa* was found fit due to the results observed in the SAM and was judged to be easy to read and thus potentially exploitable in the cancer population. To the best of our knowledge, only two studies reported an explanation for designing a PA guidebook for cancer patients [14, 16], with similar steps to the ones we adopted. Previous studies reported low levels of quality and accuracy of written PA information in cancer [21, 22]. To prevent this possibility, we have applied a rigorous methodology that permitted us to realize a guidebook, according to the recent literature in the field and the current guidelines for PA in cancer.

Regarding the readability (the ease of comprehension as a result of the writing style), the *Informa* guidebook was found to be appropriate for patients having at least a primary education for both reading formulae. Specifically, the GulpEase level was equal to 61, whereas the Flesch-Vacca index obtained a score of 66. These findings are in line with the two studies of Vallance and colleagues [14, 16], which reported a preliminary readability analysis before dissemination of the materials. Nevertheless, other studies investigating the readability of already available health-related resources for cancer patients found that most materials have a high-grade level of readability and consequently are difficult to read and understand [13, 23]. A recent study by Goodwin and colleagues assessed the prevalence, nature, and contents of the available online information regarding PA and sedentary behavior for cancer patients. A large majority of contents aiming to improve PA resulted in lacking specific recommendations and detailed advice. Indeed, if the contents highlighted the benefits of PA, less than half reported its amount and intensity as recommended by the current guidelines, whereas advices on how to improve PA were rarely included, as well as three-quarters of materials lacked information about risks and precautions of exercising. Moreover, the Flesch Reading Ease rating showed that 80% of the included websites reported an unacceptable readability score [13]. This study highlights the urgency of providing health information that is evidence-based, suitable, and readable, with the aim of offering the best approach to improve PA in cancer. Our research follows this direction and aims to respond to the gap in literature and patients’ needs. The importance of having readable materials is due to the fact that the scarce health literacy of patients is one of the most important reasons leading to a worse prognosis [24] and this point, particularly in the oncological setting, plays a crucial role. Indeed, written health materials, designed to promote health may educate and persuade cancer patients to adopt and maintain a healthy lifestyle, increase their exercise level, and therefore, potentially impact their survival, as reported in recent evidence about PA [2], but they must be adequately understood to be efficacious.

On the other hand, the suitability of the *Informa* guidebook was high at 94%, and in the six sub-areas, the guidebook was evaluated as “superior”. Only one single item, regarding the cover graphic, was found “adequate”, with a score of 62%, which suggests that its revision may be useful. Nevertheless, this result is encouraging because the majority of health materials for cancer patients were found “not suitable” or only “adequate” for the target population. Weintraub and colleagues assessed the suitability of 29 written educational materials for prostate cancer patients and found that only 20.6% of them were considered superior [25]. Another study exploring the suitability of online resources for family caregivers of cancer patients showed that none of the materials included in the analysis had a SAM score > 70 [23]. Our study reports high suitability scores for both patients and experts. The *Informa* booklet responds to specific needs. In a qualitative study exploring the factors influencing PA in oncology, cancer patients expressed that having credible and suitable information regarding PA can promote and facilitate engagement [17]. From healthcare perspectives, Cantwell and colleagues reported that lack of resources regarding PA for cancer patients, like education leaflets and materials, was considered one of the top-three barriers to promoting an active lifestyle in patients living with cancer [26].

This study has the strength that the guidebook was developed by authors affiliated with a reputable university and with publications on the topic, which consequently made the material credible and accurate. Nevertheless, some limitations should be noted. The majority of the study participants were highly educated, making the study results difficult to be generalized. On the other hand, we balanced the participants, according to previous studies [14, 16], preferring not only cancer patients but also other figures, such as medical oncologists, researchers in physical activity, kinesiologists, due to the fact that the guidebook involves stakeholders with different expertise. To our knowledge, an Italian version of the SAM questionnaire is not available. Although we used a rigorous method to translate the questionnaire, a future study should validate the SAM questionnaire among Italian speakers. We assessed the *Informa* guidebook only for its readability and suitability. Other instruments, such as the DISCERN checklist, could provide additional information about the quality and usefulness of the written material. Moreover, we can only speculate, with this preliminary data, about the effectiveness of the *Informa* guidebook. Moreover, we are planning to design a trial testing the efficacy of this guidebook on PA level of cancer patients in order to validate its impact. Finally, given the scarcity of literature on this topic, especially in Italy, it is difficult to make comparisons with other studies.

### Conclusions

Written resources are important tools with which patients can acquire information and knowledge, facilitate their learning, and increase their empowerment to make informed decisions and assume responsibility for their choices and lifestyles, if the materials are suitable and readable. Our study represents an important indication of the suitability and readability of our PA guidebook. Moreover, it provides useful information regarding designing and evaluating health written materials that may also be transferred to other health-related settings.

## Data Availability

Yes.

## Code Availability

N/A
